# Use of Pre- and Intensified Postprocedural Physiotherapy in Patients with Symptomatic Aortic Stenosis Undergoing Transcatheter Aortic Valve Replacement Study (the 4P-TAVR Study)

**DOI:** 10.1155/2021/8894223

**Published:** 2021-01-16

**Authors:** M. Weber, U. Klein, A. Weigert, W. Schiller, V. Bayley-Ezziddin, D. C. Wirtz, A. Welz, N. Werner, E. Grube, G. Nickenig, J.-M. Sinning, A. Ghanem

**Affiliations:** ^1^Department of Medicine II, Heart Center Bonn, University Hospital Bonn, Bonn, Germany; ^2^Department of Cardiac Surgery, Heart Center Bonn, University Hospital Bonn, Bonn, Germany; ^3^Clinic for Orthopedics and Trauma Surgery, University Hospital Bonn, Bonn, Germany

## Abstract

**Background:**

Physiotherapy prior to open-heart surgery lowers the rate of pneumonia and length of the hospital stay. Pneumonia is a major contributor to short-term mortality following transcatheter aortic valve replacement (TAVR). Hence, we hypothesized that pre- and intensified postprocedural physiotherapy in patients undergoing TAVR might impact the net functional and clinical outcome.

**Methods and Results:**

The 4P-TAVR study was a prospective, monocentric, randomized trial. The study was designed to compare the efficacy and safety of intensified periprocedural physiotherapy including inspiratory muscle training versus standard postprocedural physiotherapy. Patients were randomized in a 1 : 1 fashion. 108 patients were included and followed up for 90 days after TAVR. While patients in group A (control group: 50 patients, age: 81.7 ± 5.0 years, 52% male) did not receive physiotherapy prior to TAVR, group B (intervention group: 58 patients, age: 82.2 ± 5.82 years, 47% male) participated in intensive physiotherapy. Compared to the control group, patients in the interventional group showed a lower incidence of postinterventional pneumonia (10 [20.0%] vs. 3 [5.1%], *p*=0.016) and had a 3-day shorter mean hospital stay (13.5 ± 6.1 days vs. 10.1 ± 4.7 days, *p*=0.02). The primary composite endpoint of mortality and rehospitalization was not different between the groups.

**Conclusion:**

Intensified physiotherapy is safe and has positive effects on clinical outcomes up to 90 days after TAVR but has no impact on the primary combined endpoint of mortality and rehospitalization. Longer follow-up, a multicenter design, and a higher number of subjects are needed to confirm these preliminary results. This trial is registered with DRKS00017239.

## 1. Background

According to the current guidelines, postoperative physiotherapy is considered as a class I B indication after surgical aortic valve replacement (SAVR) [1]. However, evidence is mainly based on retrospective or prospective registries with a lack of randomized clinical trials. Actually, there is no evidence for the benefits of postoperative physiotherapy after transcatheter aortic valve replacement (TAVR), but a treatment similar to that after SAVR is the standard of care in most TAVR centers. Registry data demonstrated up to 30% short-term pulmonary morbidity and mortality after TAVR [2]. Whether preoperative physiotherapy, before TAVR, has useful effects is unknown. Initial data indicate that preprocedural physiotherapy, prior to open-heart surgery, significantly lowers the rate of pneumonia and the length of the hospital stay. A randomized controlled trial by Hulzebos et al. showed that preoperative physiotherapy before coronary artery bypass grafting could significantly lower the rate of pneumonia and the length of the hospital stay [3]. Hence, we hypothesized that pre- and intensified postprocedural physiotherapy in patients undergoing TAVR could affect net positive clinical benefits and outcome.

## 2. Methods

**Figure 1:**
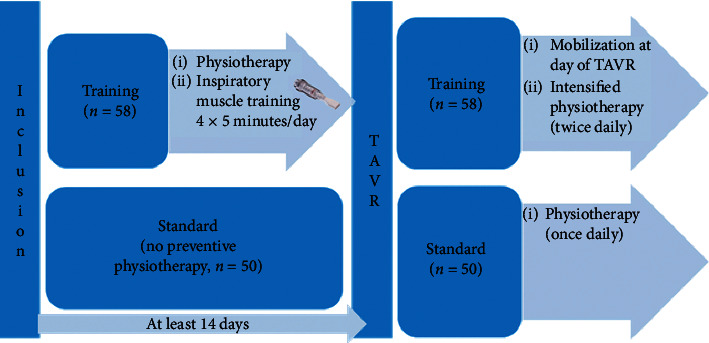
Study flow chart.

The “Use of Pre- and Intensified Postprocedural Physiotherapy in Patients with Symptomatic Aortic stenosis undergoing Transcatheter Aortic Valve Implantation Trial” (4P-TAVR study) is a prospective, randomized, open-label, controlled trial that was conducted at the Heart Center Bonn. The study was designed to compare the efficacy and safety of intensified pre- and postprocedural physiotherapy versus the standard of postprocedural physiotherapy alone. The trial was approved by the local ethics committee (022/12), and a flow chart of the study is illustrated in Figure 1. The combined primary efficacy endpoint of the 4P-TAVR study is all-cause mortality or all-cause hospitalization after 90 days. Our hypothesis was that pre- and postprocedural physiotherapy lowers the incidence of pulmonary complications and hence reduces all-cause mortality and rehospitalization. Power analysis revealed a sample size of 110 patients per group to reach a power of 0.8 and detect a 35% reduction of the primary endpoint. Secondary outcome measures include the occurrence of individual major adverse cardiovascular and cerebrovascular event (MACCE) components, incidence of pneumonia, cardiovascular mortality, and/or cardiovascular rehospitalization at 30 days and 3 months following TAVR. Pneumonia was defined as X-ray or CT scan suggestive of pneumonia and fever >38°C without other causes or leukopenia (<4.000 WBC/mm³) or leukocytosis (>12.000 WBC/mm³) and two of the following symptoms: new onset of purulent sputum, cough or dyspnea or tachypnea, suggestive auscultation (rales or bronchial breath sounds), and worsening gas exchange (e.g., O_2_ desaturation or increased oxygen requirements or increased ventilation demand).

**Figure 2:**
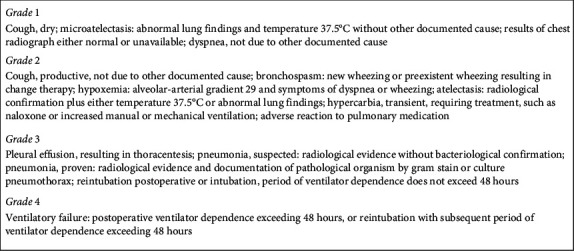
Operational definitions of postoperative pulmonary complications by Kroenke et al. [4].

Other endpoints such as pulmonary (inspiratory and expiratory capacities, chest X-ray, and incidence of postinterventional pulmonary complications (PPCs, as defined by Kroenke et al., Figure 2)) and clinical parameters (New York Heart Association Functional Classification, serial creatinine level and creatinine clearance using the Cockcroft–Gault formula, requirement for renal replacement therapy, length of intensive care unit stay, length of hospital stay, and six-minute walk test) will serve as surrogate endpoints for prognosis [4]. All parameters are following the standardized endpoint definitions of the Valve Academic Research Consortium (VARC) [5].

Besides efficacy, the trial addresses the issue of safety of pre- and postprocedural physiotherapy. Safety assessment includes mortality, myocardial infarction, stroke, bleeding of any kind, and acute kidney injury according to the VARC criteria [5]. Death is defined as death from any cause.

### 2.1. Patient Population

The study cohort consists of 108 patients with severe aortic stenosis, considered to be at high risk for surgery. Patient enrollment began in March 2012 and ended in March 2017. TAVR was performed by three experienced operators; the transcatheter heart valve (THV) used was chosen by individual anatomic patient conditions. Main inclusion criterion was severe and symptomatic aortic valve stenosis according to the existing guidelines [6–8] and comorbidities, such that one cardiologist and one cardiac surgeon agree that medical factors preclude a more-invasive valve replacement operation, based on the conclusion that the probability of death or serious morbidity exceeds the probability of meaningful improvement.

The exclusion criteria were circumstances that affected mobility, such as immobilization in a wheelchair, bedridden patients, and patients with major depressive disorders or severe dementia (resulting in either the inability to provide informed consent for the trial/procedure, the inability to maintain an independent lifestyle outside of a chronic care facility, or the likelihood that their condition would fundamentally complicate rehabilitation from the procedure or compliance with follow-up visits). Untreated, clinically significant coronary artery disease requiring revascularization or cardiogenic shock, manifested by low cardiac output, vasopressor dependence, mechanical hemodynamic support, or a need for emergency surgery, for any reason, were defined as exclusion criteria. Moreover, ongoing sepsis as well as active endocarditis or a life expectancy <12 months due to associated noncardiac comorbid conditions were not compatible with study inclusion, and these patients were excluded.

### 2.2. Randomization and Patient Treatment

Patients were randomized by using a computer-based program in a 1 : 1 ratio between one of the two treatment groups. Due to simple and no block or stratified randomization, the sample numbers can be assigned unequally. The intervention group (IMT group) received individualized ambulatory physiotherapy exercises on a daily basis for a minimum of two weeks prior to TAVR. In brief, preprocedural physiotherapy comprised inspiratory muscle training (IMT, 4 × 5 minutes/day) and a minimum of 30 minutes of walking below the threshold of subjective exhaustion. Following the transfemoral, percutaneous TAVR procedure, which was performed under conscious sedation and local anesthesia, intensified care began the same evening and comprised a mobilization protocol and individual physiotherapy with the supervision of a physiotherapist for 2 × 30 minutes per day until discharge. The control group received only postoperative physiotherapy for 1 × 30 minutes per day until discharge. All patients were monitored with a mobility tracker (SenseWear™) for counting their steps and monitoring energy turnover.

### 2.3. Statistical Analysis

Exploratory data analysis was performed, and no adjustment was made for multiple tests. A normal distribution of continuous variables was examined using the Kolmogorov–Smirnov test. Continuous data are expressed as the mean ± SD. Two-tailed *p* values were calculated and considered to be significant if <0.05. Comparisons between two groups were performed using Student's *t*-tests for paired samples or pairwise comparisons with Wilcoxon's signed-rank tests for paired continuous variables. For categorical data, Fisher's exact tests or Pearson's chi-square tests were performed.

The multivariable model was built by selecting baseline variables of clinical interest and/or satisfaction of the entry criterion of *p* < 0.05 in the univariable analysis: New York Heart Association classification, six-minute walking test distance, duration of intensive care unit stay, maximal inspiratory pressure, and procedure duration. A two-sided alpha level of 0.05 was used for all superiority testing. Statistics were performed using IBM SPSS for Windows (IBM SPSS Statistics, version 24.0.0.0; SPSS Inc., Chicago, IL).

## 3. Results

### 3.1. Clinical Outcomes and Baseline Characteristics

108 patients, which were scheduled for elective TAVR and fulfilled both the in- and exclusion criteria, were recruited at the Heart Center Bonn between May 2012 and May 2017. These patients (mean age: 82.0 ± 5.5, mean logistic EuroSCORE: 21.12 ± 14.31) were randomly assigned to the two study groups (58 were allocated to the IMT group and 50 to the standard care group). Baseline characteristics are shown in Table 1 and showed no significant differences between the groups. Planned sample size of 220 patients was not reached mostly because the mandatory training period of two weeks prior TAVR was not possible in many patients. Enrollment was stopped after 5 years.

The mean number of weeks awaiting TAVR was 4.3 ± 2.8 weeks for the IMT group and 4.2 ± 2.8 weeks for the standard care (SC) group (*p*=0.31). The duration of the TAVR procedure and the amount of contrast agent used were not significantly different between the two groups (Table 1).

### 3.2. Success of Physiotherapy and Respiratory Training

The daily inspiratory muscle workout was recorded by all participants in the intervention group in their training diaries. None of the training patients dropped out, and all participants in the IMT group returned the questionnaire (mean ± SD scores for motivation and endurance on a 4-point scale were 2.5 ± 0.9 and 2.6 ± 1.2, respectively). The subgroups were too small for an analysis of the degree of motivation regarding outcome.

Patients in the IMT group exercised for a mean of 30.2 ± 19.9 days without experiencing adverse events during or after the training sessions. The mean inspiratory muscle strength, assessed by measuring the maximal pressure at residual volume, increased significantly from 32.4 ± 15.9 cm H_2_O at baseline to 36.8 ± 18.2 cm H_2_O (*p*=0.009) at the end of the preoperative training period in the IMT group, but not in the standard care group (36.7 ± 15.8 vs. 37.9 ± 17.3 cm H_2_O, *p*=0.55). We found no differences between the two groups, neither at the time of inclusion nor prior to TAVR (Table 2).

Every patient that was included wore a mobility tracker (Sense Wear™) prior to TAVR, and this revealed a trend towards a higher step count per day among patients in the training group compared to the standard care group (1024 ± 1301 steps per day vs. 515 ± 910, *p*=0.079). The results of a six-minute walk test were not different between the two groups prior to TAVR (Table 1). The SenseWear band additionally showed a higher daily energy turnover in the training patients (7628.2 ± 2281.5 kilojoules vs. 5062.8 ± 4264.2 kilojoules, *p*=0.001), and after TAVR, the training patients received on average 6.4 ± 6.0 units of physiotherapy, whereas the control patients got 3.7 ± 3.2 physiotherapy units prior discharge (*p*=0.045).

### 3.3. Outcome Measurements

The primary outcome was defined as rehospitalization or mortality after 90 days. Of the 108 patients enrolled, 35 (32.4%) patients either died or were hospitalized during the first 90 days after TAVR. There was no significant difference between the study groups (20 (34.5%) vs. 15 (30.0%), *p*=0.44). Only the first endpoint occurring in an individual patient was counted in this analysis. Regarding the endpoints separately, no difference was found between the groups neither for mortality (6 (10.3%) vs. 5 (10.0%), *p*=0.62) nor for rehospitalization (16 (27.6%) vs. 12 (24.0%), *p*=0.30, Table 3).

**Figure 3:**
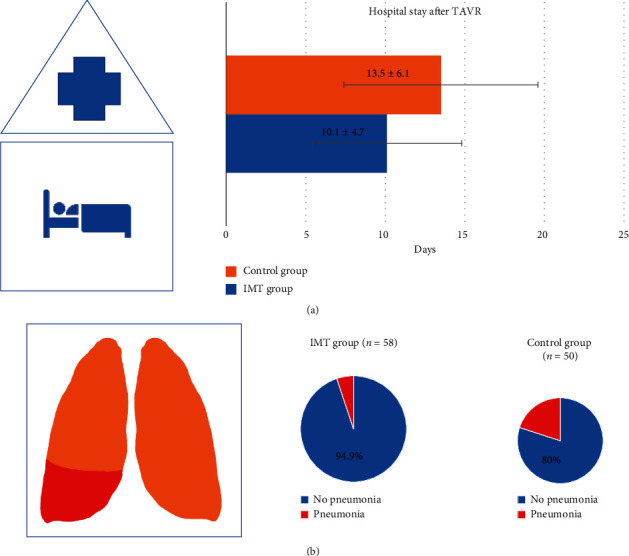
(a) Hospital stay after TAVR and comparison between groups (*p*=0.02); (b) incidence of pneumonia and comparison between IMT (left) and control (right) group (*p*=0.02).

Additionally, after 30 days, the combined MACCE endpoints (myocardial death, myocardial infarction, stroke, and major vascular complications) were not different between the two groups (Table 3). Pneumonia occurred significantly more often in the control than in the training group after TAVR (10 (20.0%) vs. 3 (5.1%), *p*=0.016, Figure 3). All pneumonic events were apparent during the first 30 days, and the bacteriological spectrum was similar in both groups. Three of 13 patients developing pneumonia died subsequently during the first 90 days, all of whom were in the control group. A total of 4 (6.9%) of the 58 patients in the IMT group and 10 (20.0%) of 50 patients in the standard care group developed a postoperative pulmonary complication (PPC) grade of at least 3 (Table 3, *p*=0.028). The mean duration of postinterventional hospitalization was 10.1 (±4.7) days in the IMT group and 13.5 (±6.1) days in the standard care group (Table 3 and Figure 3), which was significantly different (*p*=0.02). When excluding patients with pneumonia from analysis, hospital duration of both groups did not show a significant difference anymore.

To account for differences in the baseline characteristics that were evident after randomization between the groups of patients undergoing IMT and standard care, we performed two post hoc analyses. In the first analysis, we performed a stratified analysis among 23 patients with chronic obstructive lung disease (COLD) and found no significant therapeutic effect of physiotherapy with respect to pneumonia or length of hospital stay (pneumonia in the IMT group: 0 and pneumonia in the control group: 1, *p*=0.304; hospital stay: 8.7 ± 3.5 days vs. 15.1 ± 12.9 days, *p*=0.24) in these small subgroups. We also performed a logistic regression analysis looking at the relationship of potentially confounding variables (New York Heart Association classification, six-minute walking test distance, duration of intensive care unit stay, maximal inspiratory pressure, and duration of the procedure) and the primary combined outcome as well as incidence of pneumonia and found that intensive care unit stay (OR: 1.58, CI: 1.15–2.17, *p*=0.005) and maximal inspiratory pressure (OR: 0.95, CI: 0.92–0.99, *p*=0.03) were independent predictors for the combined primary endpoint (90-day mortality or rehospitalization). The only independent predictor for the incidence of pneumonia was the length of stay in the intensive care unit (OR: 1.38, CI: 1.04–1.8, *p*=0.025).

## 4. Discussion

Our study is the first randomized clinical trial on the impact of peri-interventional preventive physiotherapy in high-risk surgical patients scheduled for elective TAVR due to severe aortic stenosis. Also, the primary endpoint–composed of mortality and rehospitalization at 90 days–was not met, preinterventional physiotherapy with IMT was found to significantly improve inspiratory muscle function. Furthermore, in patients receiving perioperative physiotherapy, the incidence of pneumonia was significantly reduced by 75% compared with patients receiving standard care. Additionally, the duration of the postinterventional hospital stay was significantly reduced in the training group by 25%. The mortality and rehospitalization rates were not influenced by intensified physiotherapy.

The typical TAVR collective usually has a moderate to high-risk status and is prone to suffer from perioperative respiratory infections. Nowadays, TAVR is a highly standardized procedure with very low interventional complications. Therefore, it is important to also keep the respiratory complications as low as possible. Tirado-Conte et al. showed that when patients experience peri-interventional infections, the mortality rate rises from six to fourteen percent and the duration of hospitalization is lengthened by eight days in TAVR patients. Of such perioperative infections, respiratory infections represent the majority with 39–44% [9]. Our cohort consisted of high-risk patients with a mean logistic EuroSCORE of more than 20%. Peri-interventional physiotherapy including preinterventional inspiratory muscle training resulted in a significant improvement (14% increase) in mean inspiratory muscle strength (32.4 ± 15.9 cm H_2_O at baseline to 36.8 ± 18.2 cm H_2_O after the preinterventional training period) without causing adverse effects. These results are in line with the study results by Weiner et al. and Hulzebos et al. which involved patients undergoing coronary artery bypass grafting (CABG) surgery [3, 10]. Our data suggest that preinterventional IMT and peri-interventional physiotherapy seem to prevent the consequences of peri-interventional immobilization. Besides the duration of stay in the intensive care unit, maximum inspiratory pressure was the only predictor of the combined primary endpoint of 90-day mortality and rehospitalization. Peri-interventional physiotherapy and preinterventional inspiratory muscle training also promoted postoperative recovery because the mean duration of hospitalization was three days shorter on average in the 58 patients in the IMT group than it was in the 50 patients in the standard care group (10.1 vs. 13.5 days, *p*=0.02, Figure 3), respectively. However, total hospital stay was quite long in both groups but is in line with a recent publication of the German aortic valve registry that showed a mean length of hospital stay (admission-discharge) of 13 days in high-risk patients [11].

Despite a higher rate of pneumonia in the control group, we found no difference in mortality or rehospitalization rate between the IMT and the control group. To date, there is no randomized trial that could detect a difference in mortality or rehospitalization following intensified physiotherapy before and after TAVR. Probably, this is because the overall number of subjects in our study was too low to detect a difference in mortality.

In our study, the incidence of pneumonia was reduced by 75% in the IMT group (Figure 3) and is comparable with data of Hulzebos et al. in CABG patients (IMT group: 6.5% vs. 16.1% in the standard care group; OR 0.40; 95% CI, 0.19–0.84), who also showed a 60% reduction with IMT [3].

We found no differences in the bacterial spectrum; however, the overall event rate (*n* = 13) was low for such an analysis. Having a longer stay in the intensive care unit was shown to be an independent predictor for incidence of pneumonia. Not surprisingly, the higher incidence of pneumonia in the control group came along with higher PPC scores. These results are in line with a study by Nomori et al., who showed that IMT before surgery is able to prevent PPCs and also a study by Rajendran et al., who showed that CABG patients with preoperative short-term pulmonary rehabilitation and preexisting chronic obstructive lung disease improved their pulmonary function and decreased the incidence of atelectasis and health care expenditure as evidenced by a shorter ventilation time and shorter hospital stay [12, 13]. The concept of prerehabilitation raises hope also in oncology, as shown by Fujimoto and Nakayama in a retrospective analysis of 15,146 lung cancer patients before and after surgery [14]. They showed that the onset of pneumonia was less frequent in patients with perioperative rehabilitation. It must be stated that clinical application of a detailed prehabilitation program is dependent on the number of physiotherapists and of course a question of reimbursement.

Dysfunction of the respiratory muscles due to surgery or immobilization may lead to a reduction in vital capacity, tidal volume, and total lung capacity [15]. This can cause atelectasis in the basal lung segments and may be a risk factor for pulmonary infections, which could lead to a significant impact on morbidity and mortality in this patient population. Our cohort did not undergo surgical valve replacement, but due to advanced age and severe aortic valve stenosis, immobilization—especially after the procedure—is common. We found that preventive and intensified postinterventional physical therapy with IMT, administered before TAVR to patients at high risk of developing PPCs, was associated with an increase in inspiratory force and a decrease in the incidence of PPCs and length of hospitalization. We consider this to be an important preprocedural intervention that appears to be effective at reducing morbidity. Nowadays, TAVR patients are younger and belong mostly to the low-intermediate risk groups. These patients can be mobilized faster, and mean hospital stay length will be shorter as well. Trials with a higher number of subjects and a longer preinterventional training period as well as seamless transition to postinterventional rehabilitation therapy are needed to confirm these results. We hypothesize that the principle of “the fitter the better” also applies to TAVR patients. Recently, Abdul-Jawad Altisent et al. reported that patients with increased exercise capacity six months after a TAVR, compared to baseline, have a better prognosis than those without [16]. We are looking forward to the upcoming multicenter randomized perform TAVR trial (NCT03522454) with 220 patients randomly allocated to receive a multifaceted intervention consisting of a home-based exercise program and a protein-rich oral nutritional supplement or standard lifestyle counseling. The primary endpoint will be the change in short physical performance battery scale score, three months after the procedure. Results are not expected before 2020. To translate these results into daily practice, however, more physiotherapists, adequate reimbursement of their work, and finally, an outstanding motivation of patients and their relatives are essential. Furthermore, we need to implement a seamless transition for the patient from the hospital where the TAVR is performed to a specialized rehabilitation center. Taken together, this might be a reasonable strategy to prevent adverse respiratory events both before and after TAVR. Our results are promising and should be verified in larger multicenter and randomized trials.

## 5. Conclusion

Intensified peri-interventional physiotherapy was shown to be safe and could have positive effects on clinical outcome up to 90 days after TAVR. Due to the missing difference in the primary endpoint, the 4P-TAVR study should be regarded as preliminary and hypothesis generating. A longer follow-up is needed, together with a multicenter design and a larger sample size, to confirm these results.

## 6. Limitations

In general, our study had a few limitations, and thus, our findings may be restricted. First, the monocentric character of our study cannot proof the generalizability of the training program. For a multicenter approach, a detailed physiotherapy protocol and adequate manpower is needed to conduct this intensified peri-interventional physiotherapy. Second, planned sample size of 220 patients was not reached mostly because the mandatory two-week training period prior TAVR was not possible in many patients. Hence, the statistical power of this analysis is very limited due to a small event rate of the combined primary endpoint (*n* = 35) and the rate of pneumonia (*n* = 13). Lastly, using first- and second-generation TAVR devices and larger sheaths, treating a high-risk population could be additional confounders, whereas nowadays, most patients are at intermediate risk and are treated by third-generation devices.

## Figures and Tables

**Table 1:** Baseline parameters.

	All patients (*n* = 108)	Training group (*n* = 58)	Control group (*n* = 50)
Age (years)	82.0 ± 5.5	82.2 ± 5.8	81.7 ± 5.0
Female gender (*n* (%))	55 (50.9)	31 (53.4)	24 (48)
BMI (kg/m^2^)	26.8 ± 4.6	26.3 ± 4.7	27.4 ± 4.5
STS-PROM (%)	7.8 ± 6.1	8.5 ± 7.0	7.0 ± 4.8
Logistic EuroSCORE (%)	21.1 ± 14.3	21.5 ± 14.1	20.7 ± 14.7
NYHA class	3.1 ± 0.5	3.1 ± 0.5	3.1 ± 0.4
NYHA class >2 (*n* (%))	100 (93)	53 (91)	47 (94)
COLD (*n* (%))	23 (21)	16 (28)	7 (14)
CAD (*n* (%))	70 (64.8)	34 (58.6)	36 (72)
AF (*n* (%))	46 (42.6)	26 (44.8)	20 (40)
LVEF (%)	54.0 ± 12.9	53.5 ± 11.9	54.6 ± 14.1
Pressure mean gradient (mmHg)	40.7 ± 14.8	40.9 ± 15.7	40.4 ± 13.6
MR ≥ II° (*n* (%))	6 (6)	2 (3)	4 (8)
Pulmonary artery pressure (mmHg)	36.2 ± 18.9	35.6 ± 18.1	37.0 ± 20.0
Creatinine (mg/dl)	1.48 ± 0.82	1.58 ± 1.13	1.31 ± 0.50
NT-pro BNP (ng/ml)	4725 ± 5760	4507 ± 5182	4996 ± 6455
Baseline six-minute walk test (m)	222.1 ± 115.4	221.4 ± 109.1	223.1 ± 124.5
Procedure duration (min)	74.2 ± 34.9	71.2 ± 29.7	77.9 ± 40.5
Amount of contrast media (ml)	148.2 ± 44.8	145.3 ± 40.4	151.7 ± 49.8

BMI, body mass index; STS-PROM, Society of thoracic surgeons predicted probability of mortality score; COLD, chronic obstructive lung disease; CAD, coronary artery disease; LVEF, left ventricular ejection fraction; AF, atrial fibrillation; MR, mitral regurgitation; there were no significant differences between the groups; therefore, *p* values were omitted in the baseline characteristics due to the randomized study design.

**Table 2:** Respiratory, mobility, and training parameters.

	All patients (*n* = 108)	Training group (*n* = 58)	Control group (*n* = 50)	*p* value
MIP (inclusion, cm H_2_O ± SD)	34.2 ± 15.8	**32.4** ± **15.9**^*∗*^	36.7 ± 15.8^#^	0.73
MIP, (pre-TAVR, cm H_2_O)	37.2 ± 17.8	**36.8** ± **18.2**^*∗*^	37.9 ± 17.3^#^	0.47
FEV1 (inclusion, l/sec)	1.34 ± 1.1	1.28 ± 1.13	1.41 ± 1.06	0.53
Step count per day prior TAVR	833 ± 1189	1024 ± 1301	515 ± 910	0.079
Total energy turnover per day (kilojoule)	6670.4 ± 3380.9	7628.2 ± 2281.5	5062.8 ± 4264.2	**0.001**
Physiotherapy units post-TAVR	5.5 ± 5.4	6.4 ± 6.0	3.7 ± 3.2	**0.045**
Weekly training time prior TAVR (minutes, median, (CI))	73.0 (37.8–125.0)	105.9 (72.8–115.0)	40.2 (29.3–53.1)	**0.001**
Time until TAVR (weeks)	4.3 ± 2.8	4.3 ± 2.8	4.2 ± 2.8	0.31

MIP, maximum inspiratory pressure; FEV1, forced expiratory volume in one second; ^*∗*^*p*=0.09; ^#^*p*=0.55.

**Table 3:** Outcome measures.

	All patients (*n* = 108)	Training group (*n* = 58)	Control group (*n* = 50)	*p* value
90-day mortality (*n* (%))	11 (10.2)	6 (10.3)	5 (10.0)	0.62
90-day rehospitalization (*n* (%))	28 (25.9)	16 (27.6)	12 (24.0)	0.30
90-day mortality or rehospitalization (*n* (%))	35 (32.4)	20 (34.5)	15 (30.0)	0.44
Hospital duration (days)	11.62 ± 7.19	10.1 ± 4.7	13.5 ± 6.1	**0.023**
Duration of intensive care therapy (days)	3.2 ± 1.9	3.1 ± 1.7	3.4 ± 2.2	0.34
PPC score	0.87 ± 1.04	0.74 ± 1.00	1.02 ± 1.07	0.21
PPC score >2 (*n* (%))	14 (12.9)	4 (6.9)	10 (20.0)	**0.028**
Pneumonia (*n* (%))	13 (12.0)	3 (5.1)	10 (20.0)	**0.016**
30-day MACCE (*n* (%))	5 (4.6)	2 (3.5)	3 (6)	0.406
30-day mortality (*n* (%))	4 (3.7)	2 (3.5)	2 (4)	0.556
30-day myocardial infarction (*n* (%))	0 (0)	0 (0)	0 (0)	—
30-day major vascular complications (*n* (%))	4 (3.7)	2 (3.5)	2 (4)	0.633
30-day stroke rate (*n* (%))	1 (0.9)	0 (0)	1 (2)	0.463

PPC, postoperative pulmonary complication; MACCE, major adverse cardiac and cerebrovascular events.

## Data Availability

Data used to support the findings of this study are available upon request to the corresponding author.
